# Mussels and Local Conditions Interact to Influence Microbial Communities in Mussel Beds

**DOI:** 10.3389/fmicb.2021.790554

**Published:** 2022-01-13

**Authors:** Edward Higgins, Thomas B. Parr, Caryn C. Vaughn

**Affiliations:** ^1^Oklahoma Biological Survey and Department of Biology, University of Oklahoma, Norman, OK, United States; ^2^National Park Service, Great Lakes Inventory and Monitoring Network, Ashland, WI, United States

**Keywords:** freshwater mussel, microbiome, nutrient cycling, sulfur, ecosystem function, nitrogen

## Abstract

Microbiomes are increasingly recognized as widespread regulators of function from individual organism to ecosystem scales. However, the manner in which animals influence the structure and function of environmental microbiomes has received considerably less attention. Using a comparative field study, we investigated the relationship between freshwater mussel microbiomes and environmental microbiomes. We used two focal species of unionid mussels, *Amblema plicata* and *Actinonaias ligamentina*, with distinct behavioral and physiological characteristics. Mussel microbiomes, those of the shell and biodeposits, were less diverse than both surface and subsurface sediment microbiomes. Mussel abundance was a significant predictor of sediment microbial community composition, but mussel species richness was not. Our data suggest that local habitat conditions which change dynamically along streams, such as discharge, water turnover, and canopy cover, work in tandem to influence environmental microbial community assemblages at discreet rather than landscape scales. Further, mussel burrowing activity and mussel shells may provide habitat for microbial communities critical to nutrient cycling in these systems.

## Introduction

Key ecosystem processes are carried out by both microbes and animals, but microbial communities are particularly important to evaluate in tandem with animal influences on ecosystem function as microbiome data combined with environmental data improve our understanding of ecosystem processes (Graham et al., 2016). Further, effects of animals on microbial communities are important and underexplored (Skelton et al., 2017; Fitzpatrick et al., 2018; Thoemmes and Cove, 2020). As more systems are investigated, the implications of animal-microbial interactions and their impact on ecosystem function become more apparent. For example, marine birds translocate nutrients from the ocean to islands, where those nutrients increase organic matter decomposition rates by soil bacteria (Fukami et al., 2006); earthworms affect the function, but not community composition, of methanotrophic bacteria in landfills (Héry et al., 2008); and marsupial burrowing activity causes successional shifts in microbial community composition and increases nitrogen availability in soils (Eldridge et al., 2015). It is particularly important to understand baseline interactions between animals and microbes in the wake of climate and land use change. In this context, streams are a good study system because they are globally threatened by pollution and climate change (Jury and Vaux, 2005).

Within these systems, freshwater mussels (bivalve mollusks in the order Unionida) are large (∼10–100 mm adult shell length), long-lived (∼10 to over 100 years) benthic animals that perform important ecosystem functions in streams, such as filtering the water and recycling and storing nutrients (Vaughn and Hoellein, 2018). Freshwater mussels are globally imperiled and as mussel communities shift and populations decline (Spooner and Vaughn, 2008; Vaughn et al., 2015), evaluating mussel-microbiome interactions is critical to predicting changes in ecosystem function. Mussels often occur as dense (∼10–100 mussels/m^2^), multispecies aggregations called mussel beds and can comprise a significant portion of benthic biomass (Vaughn and Spooner, 2006). These communities can have large impacts on both biotic and abiotic factors in streams (Vaughn and Hoellein, 2018).

Of importance to stream microbial function, filter-feeding mussels burrow in the sediment and transform as well as transport organic matter from the water column into the sediment *via* excretion and biodeposition of feces and pseudofeces (rejected particles encapsulated in mucus and expelled before ingestion). We know mussel beds can significantly influence nutrient cycling in the sediment on an ecosystem-wide scale (Hoellein et al., 2017; Nickerson et al., 2019) indicating interactions with sediment microbiomes. Aquatic sediment is a unique environment in which the interface between an oxygenated surface and an anoxic subsurface microhabitat is relatively shallow and mussel burrowing activity can directly influence the microhabitats of both layers, often introducing oxic microniches into anoxic habitats (Brune et al., 2000). Levels of oxygenation can affect microbial community composition and function (Sinsabaugh et al., 2009) and benthic organisms can couple microbially driven biogeochemical processes (nitrification-denitrification, elemental sulfur cycling, etc.; Nickerson et al., 2019). However, incorporating drivers of benthic microbial community structure and diversity into riverine ecosystem function requires further research (Zeglin, 2015).

Here we consider the mussel microbiome to be comprised of the microbial communities on mussel shells and in their biodeposits. How these communities interact with the sediment microbial communities, and potential differences between mussel species in how this occurs, is a key research gap. Host physiology and diet are known to impact hosted microbiomes in a variety of organisms (Turnbaugh and Gordon, 2009; Phillips et al., 2012; Ye et al., 2014; Pierce et al., 2016) and freshwater mussels have species-specific physiological and behavioral traits (Haag, 2012). Interspecific differences may influence the mussel microbiome and therefore interactions with the sediment microbiome. For example, mussel species investigated in southeastern Oklahoma U.S. are either thermally sensitive (e.g., *Actinonaias ligamentina)* or thermally tolerant (e.g., *Amblema plicata)*, and thermally sensitive species excrete nutrients at higher rates at warm temperatures, with different stoichiometric ratios when stressed (Spooner and Vaughn, 2008). Additionally, Allen and Vaughn (2009) found that mussels exhibit species-specific differences in burrowing activity with thermally sensitive species demonstrating higher activity.

Here we asked, how similar are the freshwater mussel microbiome and the sediment microbiome, and how do these relationships change with mussel abundance, species composition, and environmental conditions? We addressed these questions with a field study comparing benthic microbiomes in three mussel beds in a small river in the southern US focusing on two dominant mussel species. We sampled microbial communities from four microhabitats: the surface layer of sediment, sediment from 6 to 10 cm below the surface, mussel shells, and mussel biodeposits. Microbes were identified using 16S rRNA analysis. We predicted that environmental conditions among microhabitats would be sufficiently distinct to host unique assemblages of microbes. We expected that differences in mussel species’ behavior and nutrient excretion would produce species-specific host-associated microbial community composition. We also expected that microbial community structure within the sediment would reflect mussel-associated changes in biogeochemical cycling.

## Materials and Methods

### Study Area and Focal Mussel Species

We conducted our study in the Kiamichi River, Oklahoma, a well-studied stream in the southcentral U.S. known for its high freshwater mussel biodiversity (Matthews et al., 2005). Mussel assemblages in this river are typically dominated by two species, *Actinonaias ligamentina* and *Amblema plicata*, that make up ∼70% of mussel biomass in this region but differ morphologically, behaviorally, and physiologically (Vaughn, 2010; Hopper et al., 2019). *Amblema plicata* has a ridged shell and tends to be sedentary, while *A. ligamentina* is an active burrower with a smooth shell (Allen and Vaughn, 2009). The two species also differ in their thermal preferences, which influences filtration rates as well as nutrient excretion rates and stoichiometry (Spooner and Vaughn, 2008; Trentman et al., 2018).

### Field Study

**FIGURE 1:**
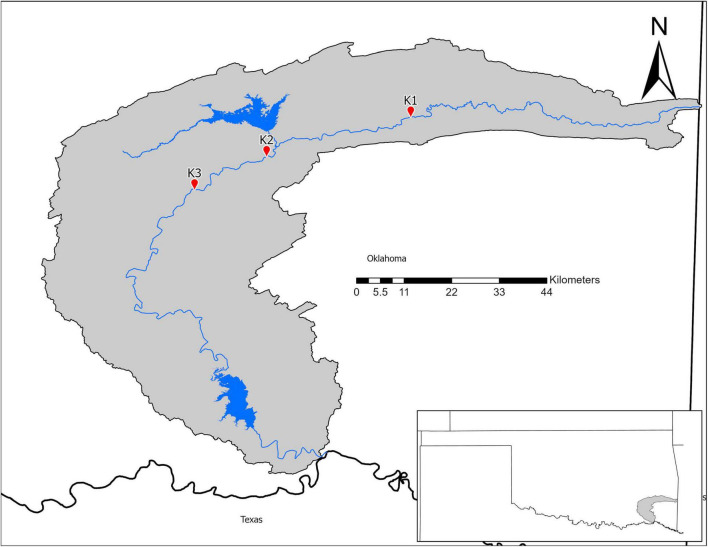
Map of the Kiamichi River drainage in Southeastern Oklahoma.

In July 2018, we collected microbial samples from mussel beds in the Kiamichi River. We selected three sites (Figure 1) with previously documented abundant, diverse mussel assemblages and data on mussel roles in nutrient recycling and storage (Atkinson et al., 2013; Atkinson and Vaughn, 2015; Hopper et al., 2018). Sites varied in abiotic characteristics that may influence environmental microbiomes such as flow, substrate, and shade. Thus, we characterized sites by measuring flow (with a Hach LDO meter), sediment particle sizes (with Wolman pebble counts), and shading (using a densiometer) in summers 2015–2016 as part of a larger study (Hopper et al., 2018; Vaughn et al., 2021).

At each of the three selected sites, we sampled four individuals of both *A. plicata* and *A. ligamentina*. For each mussel bed, we first conducted tactile searches on the sediment surface to locate mussels. Searches were conducted from downstream to upstream to minimize disturbance of the sediment. We then placed a 0.25 m^2^ quadrat around locations that contained at least one individual of each species. Although quadrats could contain multiple individuals of each species, they all contained both focal species, and we only sampled one individual of each species per quadrat. We had a total of 12 sampling locations and 24 total mussels (12 *A. plicata* and 12 *A. ligamentina*) across all sites. We used a custom 4.9 cm diameter, clear acrylic sediment corer to collect one sediment core from each quadrat, for a total of 12 sediment cores. To prevent cross-contamination between samples, we used 90% ethanol to rinse the interior of our corer between samples. Mussels have been shown to affect bacterial growth and metabolism at depths of 6 cm or greater below the surface (McCall et al., 1986; Black and Just, 2018) so we extruded the sediment from the corer with a rubber stopper and took subsamples from the surface layer (*n* = 12) and 6–10 cm below (*n* = 12) using an ethanol rinsed, flame sterilized spatula.

We removed mussel individuals from the sediment and used sterile razor blades to collect a single biofilm sample from the shell of each mussel. Blades were rinsed with 90% ethanol, wiped with a sterile kimwipe, and flame sterilized for 1 min between samples (Horton et al., 2019; Miller-ter Kuile et al., 2021). Five shell samples did not successfully sequence (three from *A. ligamentina* and two from *A. plicata*) resulting in a final n of 19. We then gently scrubbed mussel shells using sterile nylon mesh to remove the remaining biofilm and left them in containers with 1 L of filtered river water for 4–6 h to allow time for mussels to biodeposit sufficient material for collection. Biodeposits were collected using an ethanol rinsed, flame-sterilized spatula. Similarly, not all biodeposits samples sequenced successfully (2 from each species) for a final n of 20. After mussels were removed from the sediment, quadrats were excavated to a depth of 15 cm and any additional mussels were identified to species and counted (Vaughn et al., 1997). While storage at –80°C is considered optimal for microbial community samples, short term cold storage demonstrates little change in fecal and soil microbiome community structure (Rubin et al., 2013; Choo et al., 2015), and so all microbiome samples were placed in sterile cryovials, stored on ice in coolers on the shaded riverbank for no more than 5 h, and then placed in liquid nitrogen until transfer to a –20°C freezer within 4 days.

### Amplicon Library Construction and Sequencing

All samples were thawed, spun at 10,000 × gravity for 2 min, and water was removed *via* pipette. DNA was extracted using DNeasy PowerSoil^®^ kits (Qiagen, Hilden, Germany). We amplified the v4 region of the 16s rRNA gene using primers and PCR protocols from Kozich et al. (2013). We purified post PCR samples with Ampure XP beads (BeckmanCoulter, Indianapolis, IL, United States) at 1 × concentration, quantified with a Qubit Fluorometer (Thermo Fisher Scientific, Waltham, Massachusetts, United States), diluted with lab grade water to 4 nM equimolar concentrations, and pooled. Library preparation was performed at the Sam Noble Oklahoma Museum of Natural History and library sequencing was performed at the University of Oklahoma Consolidated Core Lab using 2 × 250 bp paired-end sequencing on an Illumina MiSeq.

### Bioinformatics and Data Analyses

Sequencing reads were merged and filtered using the program “AdapterRemoval” (Lindgreen, 2012). We performed closed reference OTU picking using “uParse” (Edgar, 2013) at 97% sequence similarity and assigned taxonomy with the SILVA reference database (v.32, Quast et al., 2013). After filtering out read abundances less than 0.1% of the average sequencing depth, we quantified richness and evenness of our samples with the number of unique OTUs and the Berger-Parker Dominance Index, respectively, using Quantitative Insights into Microbial Ecology (QIIME; Berger and Parker, 1970; Bolyen et al., 2019). We used Kruskal-Wallis tests to determine statistical differences in richness and evenness using the base R software (R Core Team, 2020). Significant results were further examined using Holm adjusted pairwise-Wilcoxon Rank Tests between microhabitats (Wright, 1992).

To quantify differences in beta diversity we calculated an Aitchison distance matrix (Euclidean distance of centered log-ratio transformed OTU counts) in R using the Compositions and Vegan packages (van den Boogaart and Tolosana-Delgado, 2008; Filzmoser et al., 2010; Gloor et al., 2017; Quinn et al., 2018; Oksanen et al., 2020). We used PERMANOVA to determine differences in bacterial community structure and permdisp to determine differences in dispersion among all microhabitats using Vegan’s “*adonis”* and “*betadisper”* functions, respectively. We conducted *post hoc*, pairwise PERMANOVA to evaluate differences among mussel and sediment microhabitats using the “*pairwise.adonis*” test from the pairwiseAdonis package (Wright, 1992; Martinez, 2020). We then tested the effects of site, sediment layer, mussel abundance and mussel richness on sediment microbial community structure as well as the effects of site and mussel species on shell and biodeposit microbial community structure. For both models, we used the “adonis2” function in Vegan for which the relative importance of each term is indicated by an *R*^2^ value (McArdle and Anderson, 2001; Oksanen et al., 2020). Differences in dispersion for these models were tested with Holm adjusted “*betadipser”* calculations. Environmental variables measured at each site (Ratio of D60 to D10 Wolman pebble counts, average discharge, average canopy cover, and average turnover) were correlated with bacterial communities using the “*envfit”* program in vegan.

To visualize differences in community structure, we performed principal coordinates analyses (PCA) using Aitchison distance matrices (Gloor et al., 2017). We were interested in microbial community patterns in each microhabitat, so in addition to our entire dataset we generated individual PCAs for sediment, shell, and biodeposit communities. Then to examine taxa contributing to differences in microbial community structure, we calculated axis loadings of each PCA by calculating Pearson rank-sum correlations between axis scores and CLR transformed abundances using R (Comrey and Lee, 1992; Quinn et al., 2018). Loadings with absolute *r*-values ≥ 0.70 were considered sufficiently correlated to evaluate (Comrey and Lee, 1992; Curry and Patten, 2014; Weingarten et al., 2019). We interpreted correlations on both the first and second PC axis. With this method, significant correlations with an *r*-value above 0.70 are interpreted such that higher *r*-values indicated a given taxa had higher abundances as PC values increase and *r*-values below –0.70 are interpreted as taxa demonstrating higher abundances as PC values decrease. Further, we were interested in functional differences between microbial communities and so we only interpreted taxa identified to family as higher classifications tended to encompass taxa with broad metabolic and niche preferences.

## Results

**FIGURE 2:**
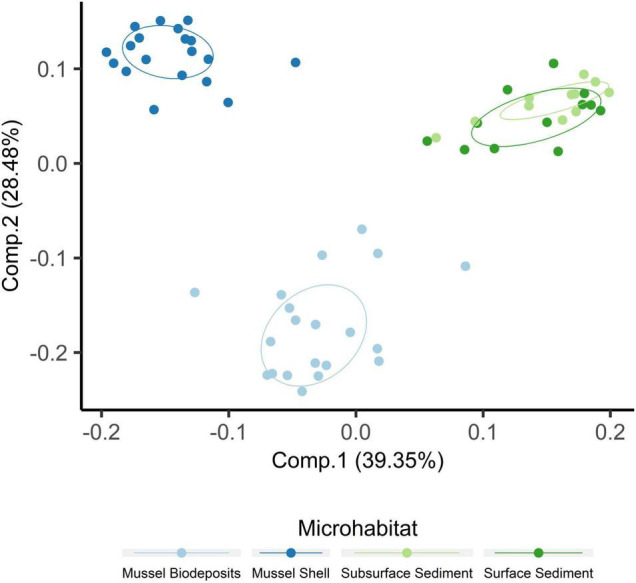
Principal coordinates analysis including samples from all microhabitats. Ellipses are drawn around centroids with a 50% confidence interval.

**FIGURE 3:**
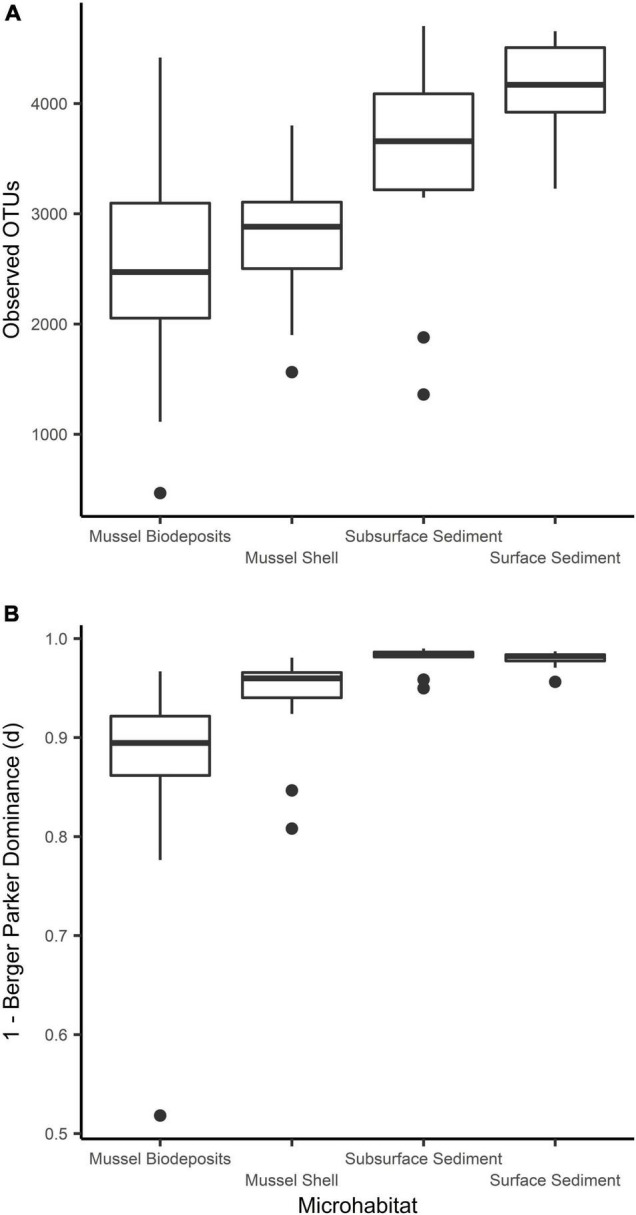
Comparisons of microbial community **(A)** richness and **(B)** evenness among sampled microhabitats.

Microhabitats displayed significant differences in microbial richness (χ^2^ = 24.65, *p* < 0.001) and evenness (χ^2^ = 40.23, *p* < 0.001; Figure 2). *Post hoc* Wilcoxon rank-sum tests showed that these differences were likely driven by mussel biodeposit and shell samples that were 44% less rich (*W* = 117.5, *p* < 0.001) and 8% less even (*W* = 811, *p* < 0.001) than sediment samples. Pairwise comparisons further showed that mussel shell and mussel biodeposit microbial communities were not significantly different in richness (*p* = 0.31), but biodeposit samples were significantly less even (*p* < 0.001). The top layer of sediment had no significant differences in richness from lower layers (*p* = 0.32) or evenness (*p* = 0.31). Bacterial community structure (*F* = 13.71, *p* = 0.001) and dispersion (*F* = 3.74, *p* = 0.016) were significantly distinct among the four microhabitats (Figure 3). Pairwise PERMANOVA demonstrated that all microhabitats were significantly different from each other (Table 1). Axis loading calculations resulted in 15 taxa with identified genera and 28 unique families significantly correlated with either the first or second axis (Table 2).

**TABLE 1:** Pairwise PERMANOVA results comparing microbial communities among microhabitats with Holm adjusted *p*-values.

Comparison	Df	Sums of squares	F-model	R2	Adjusted *p*-value
Mussel biodeposit vs. Mussel shell	1	9027.225	17.38	0.32	**0.006**
Mussel biodeposit vs. Surface sediment	1	6428.088	13.58	0.31	**0.006**
Mussel biodeposit vs. Subsurface sediment	1	6710.488	13.93	0.32	**0.006**
Mussel shell vs. Surface sediment	1	7352.131	14.43	0.33	**0.006**
Mussel shell vs. Subsurface sediment	1	7736.168	14.90	0.35	**0.006**
Surface sediment vs. Subsurface sediment	1	1649.57	3.64	0.15	**0.006**

*Statistically significant and interpreted values in bold.*

**TABLE 2:** Axis loadings for PCA comparing sediment, shell, and biodeposit microbial communities.

Family	Genus	Axis 1 r	Axis 2 r
Rhodobacteraceae	Rhodobacter	**–0.82**	0.06
Xanthomonadaceae		**–0.76**	0.34
Xenococcaceae		**–0.76**	0.42
Rhodobacteraceae		**–0.75**	0.34
Sphingomonadaceae	Novosphingobium	**–0.73**	0.14
Gemmataceae		**–0.72**	0.50
Synechococcaceae	Synechococcus	–0.49	**–0.71**
Synechococcaceae	Synechococcus	–0.45	**–0.77**
Verrucomicrobiaceae	Prosthecobacter	–0.41	**–0.77**
Synechococcaceae	Synechococcus	–0.41	**–0.80**
Synechococcaceae	Synechococcus	–0.39	**–0.81**
Chthoniobacteraceae	CandidatusXiphinematobacter	–0.37	**–0.84**
Planctomycetaceae	Planctomyces	–0.32	**–0.87**
Thermaceae	Meiothermus	–0.31	**0.71**
Pirellulaceae		–0.30	**–0.86**
Gemmatimonadaceae	Gemmatimonas	–0.29	**–0.72**
Chitinophagaceae	Sediminibacterium	–0.27	**–0.80**
Comamonadaceae		–0.25	**–0.72**
Chitinophagaceae		–0.23	**–0.88**
Pirellulaceae		–0.22	**–0.82**
Rhodocyclaceae		–0.19	**–0.86**
Fusobacteriaceae		–0.14	**–0.77**
Acetobacteraceae		–0.14	**–0.78**
Synechococcaceae	Synechococcus	–0.14	**–0.91**
Synechococcaceae	Synechococcus	–0.09	**–0.81**
Acetobacteraceae		–0.07	**–0.81**
Armatimonadaceae		–0.03	**–0.85**
Chitinophagaceae		–0.01	**–0.82**
Isosphaeraceae		0.01	**–0.73**
Bryobacteraceae		0.01	**–0.77**
Pirellulaceae		0.02	**–0.84**
Synechococcaceae	Synechococcus	0.06	**–0.83**
Caulobacteraceae	Phenylobacterium	0.06	**–0.74**
Desulfobacteraceae	Desulfococcus	**0.70**	–0.02
Thermodesulfovibrio naceae	HB118	**0.70**	0.09
Syntrophobacteraceae	Syntrophobacter	**0.71**	0.12
Myxococcaceae	Anaeromyxobacter	**0.71**	–0.02
Myxococcaceae	Anaeromyxobacter	**0.72**	0.03
Myxococcaceae	Anaeromyxobacter	**0.73**	0.07
Thermodesulfovibrionaceae	GOUTA19	**0.74**	–0.10
Desulfarculaceae		**0.76**	0.10
Thermodesulfovibrionaceae		**0.76**	0.17
Thermodesulfovibrionaceae	GOUTA19	**0.78**	0.08
Myxococcaceae	Anaeromyxobacter	**0.79**	0.19
Syntrophaceae		**0.89**	–0.03

*Higher r-values correlated with axis 1 suggest taxa associated with sediment and lower values with mussel shells. Lower r-values correlated with axis 2 will correlate with taxa differentiating mussel biodeposits from shell and sediment communities. Statistically significant and interpreted values in bold.*

**FIGURE 4:**
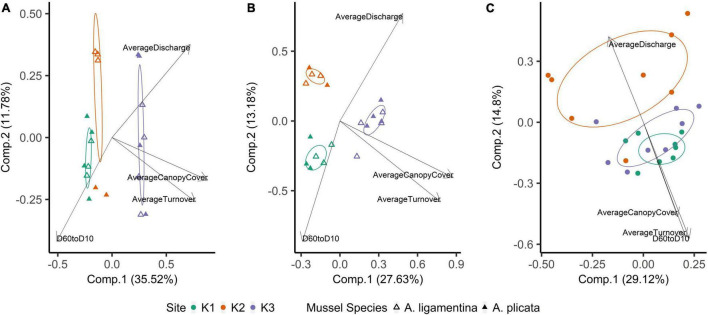
Principal coordinates analysis of **(A)** mussel biodeposit microbial communities, **(B)** mussel shell microbial communities, and **(C)** sediment microbial communities. Communities are colored based on the site from which they were sampled. Ellipses are drawn around centroids with a 50% confidence interval. Vectors were added *post hoc* and are based on *envfit* analysis.

Within quadrats, mussel abundance ranged from 3 to 13 mussels while richness ranged from 1 to 6 species. The PERMANOVA model testing the effects of site, sediment layer, mussel richness, and mussel abundance on sediment community structure was a statistically significant fit to these data (*F* = 2.52, *p* < 0.001). Of these variables, the strongest driver of sediment microbial community structure was sediment layer, followed by site, and then mussel abundance (Table 3). Mussel richness was not a significant predictor of sediment community structure (*F* = 1.144, *p* = 0.11). Sediment layer (*F* = 0.030, *p* = 0.86), mussel abundance (*F* = 1.65, *p* = 0.58), and mussel richness (*F* = 1.69, *p* = 0.58) did not show significant differences in dispersion and sites were only marginally significantly different in dispersion (*F* = 4.76, *p* = 0.07; Figure 4A). Sediment axis loadings resulted in 7 identified genera and 17 unique families (Table 4).

**TABLE 3:** PERMANOVA outputs for sediment, shell, and biodeposit models.

Model	Variable	*R* ^2^	F	*p*-value
Sediment	Sediment layer	0.15	4.49	**0.001**
	Site	0.15	2.23	**0.002**
	Mussel abundance	0.05	1.6	**0.047**
	Mussel richness	0.05	1.14	0.11
Shell	Site	0.31	3.7	**0.001**
	Mussel species	0.05	1.22	0.19
Biodeposit	Site	0.31	3.9	**0.001**
	Mussel species	0.047	1.19	0.22

*Statistically significant results in bold. Higher R^2^ values indicate more variance is explained by a given variable.*

**TABLE 4:** Axis loadings for PCA sediment microbial communities.

Family	Genus	Axis 1 r	Axis 2 r
Methanomassiliicoccaceae		**0.90**	0.31
Thermodesulfovibrionaceae	GOUTA19	**0.77**	–0.30
Thermodesulfovibrionaceae	LCP	**0.76**	–0.01
Xenococcaceae		**0.74**	–0.10
Syntrophaceae		**0.72**	–0.19
Isosphaeraceae		**0.72**	–0.32
Comamonadaceae		**–0.87**	0.08
Rhodocyclaceae		**–0.84**	0.07
Rhodocyclaceae	Dechloromonas	**–0.83**	0.13
Synechococcaceae	Synechococcus	**–0.79**	0.02
Desulfuromonadaceae		**–0.78**	–0.10
Geobacteraceae	Geobacter	**–0.78**	–0.27
Cytophagaceae		**–0.76**	0.23
Alcaligenaceae		**–0.76**	–0.23
Comamonadaceae		**–0.74**	–0.16
Chitinophagaceae		**–0.74**	0.01
Rhodocyclaceae		**–0.74**	0.05
Rhodobacteraceae	Rhodobacter	**–0.72**	–0.17
Cytophagaceae		**–0.72**	0.19
Sphingomonadaceae	Novosphingobium	**–0.70**	–0.40
Comamonadaceae		**–0.70**	–0.05
Myxococcaceae	Anaeromyxobacter	–0.07	**–0.79**
Crenotrichaceae	Crenothrix	–0.17	**–0.79**
Comamonadaceae		–0.08	**–0.70**

*Higher r-values correlated with axis 1 suggest taxa associated with subsurface sediments while lower values suggest taxa associated with surface sediments. Lower r-values correlated with axis 2 may suggest values associated with the site K1. Statistically significant and interpreted values in bold.*

Overall, the mussel biodeposit (*F* = 3.01, *p* = 0.001) and shell (*F* = 2.87, *p* = 0.001) models were statistically significant fits to these data. Mussel biodeposit microbial community structure seems to be driven by site, but mussel species was not significant (Table 3 and Figure 4B). Differences in dispersion were significant based on site (*F* = 5.75, *p* = 0.0248) but not mussel species (*F* = 0.201, *p* = 0.659). Similar to biodeposits, shell microbial communities seem driven by site, but not species (Table 3 and Figure 4C). There were no significant differences in dispersion by either site (*F* = 2.16, *p* = 0.296) or mussel species (*F* = 0.059, *p* = 0.811). Axis loadings for shell communities resulted in 8 identified genera and 18 unique families while biodeposit communities resulted in 4 identified genera and 16 unique families (Tables 5, 6).

**TABLE 5:** Axis loadings for PCA biodeposit microbial communities.

Family	Genus	Axis 1 r	Axis 2 r
Synechococcaceae	Synechococcus	**–0.96**	–0.24
Synechococcaceae	Synechococcus	**–0.89**	–0.24
Synechococcaceae	Synechococcus	**–0.86**	–0.21
Fusobacteriaceae		**–0.85**	–0.21
Rhodospirillaceae		**–0.82**	–0.40
Synechococcaceae	Synechococcus	**–0.82**	–0.34
Armatimonadaceae		**–0.77**	–0.41
Enterobacteriaceae		**–0.74**	–0.13
Synechococcaceae	Synechococcus	**–0.73**	–0.41
Chitinophagaceae		**–0.73**	–0.29
Acetobacteraceae		**–0.73**	–0.38
Sinobacteraceae		**–0.71**	0.28
Pirellulaceae		–0.34	**–0.80**
auto67_4W		–0.25	**0.73**
Pirellulaceae		–0.19	**–0.78**
Syntrophaceae		–0.16	**0.84**
Desulfobacteraceae	Desulfococcus	–0.10	**0.73**
Caldilineaceae		–0.04	**–0.70**
Sinobacteraceae	Steroidobacter	0.16	**–0.76**
Thermodesulfovibrionaceae		0.24	**0.86**
Desulfobacteraceae	Desulfococcus	**0.72**	–0.08
Chthoniobacteraceae	CandidatusXiphinematobacter	**0.87**	–0.05
Nostocaceae	Dolichospermum	**0.89**	–0.21
Chthoniobacteraceae	CandidatusXiphinematobacter	**0.94**	–0.15

*Higher r-values correlated with axis 1 suggest taxa associated with the site K3 while lower values suggest taxa associated with K2 and K1. Statistically significant and interpreted values in bold.*

**TABLE 6:** Axis loadings for PCA comparing shell microbial communities.

Family	Genus	Axis 1 r	Axis 2 r
Sphingomonadaceae		**–0.83**	–0.19
Thermaceae	Meiothermus	**–0.81**	0.00
Kouleothrixaceae	Kouleothrix	**–0.77**	0.20
Chitinophagaceae		**–0.76**	0.18
Planctomycetaceae	Planctomyces	**–0.76**	–0.19
Sphingomonadaceae	Kaistobacter	**–0.74**	0.28
Hyphomicrobiaceae		**–0.72**	0.01
Syntrophobacteraceae		–0.43	**0.73**
Chitinophagaceae		–0.26	**0.73**
Sinobacteraceae	Steroidobacter	–0.25	**0.76**
Syntrophaceae		–0.08	**–0.70**
Hyphomicrobiaceae	Hyphomicrobium	**0.70**	–0.15
Rivulariaceae	Calothrix	**0.71**	–0.18
Synechococcaceae	Synechococcus	**0.72**	0.34
Synechococcaceae	Synechococcus	**0.73**	0.24
Synechococcaceae	Synechococcus	**0.73**	0.34
Clostridiaceae		**0.74**	0.03
Sinobacteraceae		**0.75**	0.13
Synechococcaceae	Synechococcus	**0.76**	0.25
Nostocaceae	Dolichospermum	**0.76**	0.00
Hyphomicrobiaceae	Hyphomicrobium	**0.76**	0.08
Caldilineaceae	Caldilinea	**0.77**	–0.31
Comamonadaceae		**0.77**	0.38
Chthoniobacteraceae	CandidatusXiphinematobacter	**0.77**	0.08
Acetobacteraceae		**0.78**	–0.10
Synechococcaceae	Paulinella	**0.79**	0.35
Hyphomicrobiaceae		**0.79**	–0.02
Nostocaceae		**0.81**	–0.18
Caldilineaceae		**0.85**	–0.17
Acetobacteraceae		**0.86**	–0.07
Sinobacteraceae		**0.88**	0.25
Oscillochloridaceae	Oscillochloris	**0.97**	0.00

*Higher r-values correlated with axis 1 suggest taxa distinguishing the sites K1 and K2 from K3 while lower indicates the opposite. Higher r-values correlated with axis two suggest taxa distinguishing sites K2 from K3 to K1. Statistically significant and interpreted values in bold.*

The K2 mussel bed had larger substrates and pebble sizes that were evenly distributed indicated by a low D60/10. In comparison, the substrates of K1 and K3 were smaller but less evenly distributed. Discharge measurements suggest that the mussel bed at K3 typically had longer water turnover times (Table 7).

**TABLE 7:** Wolman pebble counts, substratum heterogeneity (D60/D10), flow measurements, and canopy cover collected in 2016, from Vaughn et al. (2021).

	K1	K2	K3
D10	0.13	22.1	0.18
D50	34.17	86.9	22.78
D60	51.25	115.33	28.09
D90	112	225	58
D60/D10	861.54	5.22	156.06
Average discharge (m^3^/s)	0.25	0.33	0.34
Average turnover (s)	58.62	15.47	105.69
Average canopy cover (%)	19.83	16.37	27.36

## Discussion

Our investigation revealed that the microbial communities hosted by freshwater mussels are distinct from those of the surrounding sediment. These microhabitats are in constant contact; mussels deposit feces and pseudofeces directly into the sediment and we collected biofilm from shells that were exposed to surface and subsurface sediment. Yet, sediment communities demonstrated higher alpha diversity than those that were mussel-associated, and we also found low overlap in microbial community composition among animal-associated and environmental microhabitats. Our data indicate that both environmental conditions specific to locations along the river and animal activity shape these microbial communities. Interactions relevant to critical ecological function between these microhabitats can be inferred by examining the ecology of taxa that distinguish these distinct communities.

### Site Characteristics Supersede Mussel Species Identity, Abundance, and Richness as a Driver of Microbial Community Composition Within Mussel Beds

While Weingarten et al. (2019) found that microbial communities *retained* by freshwater mussels were influenced by species as well as site, our study found that microbes on the shell and in material passed through the gut, did not differ between our focal species. These results are complementary. Much of the phytoplankton, detritus, and bacteria filtered by mussels survives gut passage alive and undamaged (Vaughn et al., 2008) and so it is possible that taxa retained in the gut may be influenced by mussel species, but taxa that pass through the gut are not. Mussel biodeposits, regardless of species, may reflect the same background food sources and differences in biodeposit community composition may be minimal when occupying the same site. The algae and bacteria able to colonize shells may similarly be site specific and this signal may overwhelm any differences in community on the basis of different shell characteristics between species.

In contrast to species, site was a strong predictor of microbial community composition in every model tested. K1 and K3 sediment communities are more similar to each other than to K2 (Figure 4C) and mussel shell and biodeposit communities at K2 and K1 are more similar to each other than to K3 (Figures 4A,B). These patterns are not entirely expected based on scale and hydrology. If increasing spatial scale were to predict our microbial community assemblages as it can in soil (Averill et al., 2021), then we would expect sites closer together to show greater overlap in community composition yet our most distant sites (K1 and K3) are more similar to each other than to K2 (Figure 1). Additionally, K1 is located upstream of a tributary impoundment, while K2 and K3 are downstream. Lack of releases from this impoundment during recent severe drought years has led to patchy drying of the lower river and increased water temperatures in shallow areas which has led to mussel declines and changes in mussel community composition (Atkinson et al., 2014; Vaughn et al., 2015). Based on these changes in flow regimes and mussel communities, these results are similarly unexpected.

Local characteristics at the stream reach level may offer insight into differences in these microbial communities. Sediment depth and particle size are both significant predictors of microbial community structure in streams (Sliva and Williams, 2005). K2’s relatively larger but more evenly distributed sediment particle sizes may drive distinct microbial communities from K1 and K3. We see this reflected in our *envfit* results which indicate higher values as community compositions grow more distinct from those of K2 (Figure 4 and Table 6). Similarly, differences in canopy cover govern shading and thus influence photosynthetic organisms on shells. K3 has the highest canopy cover and contains shell microbial communities that are most distinct from K2 and K1. K3 also has the greatest water turnover time which may partially explain its distinction in biodeposit community composition. Slower turnover in the water column will increase the duration of seston delivery which impacts what seston mussels filter (Byllaardt and Ackerman, 2014; Mistry and Ackerman, 2018), and therefore egest as biodeposits, as well as impact which bacterial taxa can colonize shells.

Mussel abundance also significantly affected sediment microbial communities. This result is supported by the findings of Black et al. (2017). They investigated relationships between sediment microbial communities and the presence or absence of mussels in the upper Mississippi River and found that sediment below mussels hosted distinct microbial communities. However, our results may underestimate the impact of mussel beds on sediment community structure. Our sampling resulted in a range of 12–56 mussels per m^2^ and unionid mussel abundances in this system can reach up to 100 mussels per m^2^. Investigating mussel impacts on sediment communities when present at higher densities may demonstrate greater significance.

### Interactions Between the Distinct Microbial Communities Found Within Benthic Microhabitats May Be a Driver of Ecosystem Function

Our data suggests that as a system, mussel shells, biodeposits, and the surrounding sediment contain microbial communities that work synergistically across microhabitats to cycle sulfur and nitrogen in aquatic environments. Interactions between mussels and the surrounding sediment is particularly relevant to ongoing investigations of the impact of freshwater mussels on ecosystem function (Vaughn and Hoellein, 2018). Studies on mussel-driven changes in nutrient cycling often focus either on the nutrients mussels cycle themselves (Atkinson and Vaughn, 2015; Trentman et al., 2018) or on ecosystem processes carried out by sediment microbial heterotrophs (Black and Just, 2018; Nickerson et al., 2019).

Across microhabitats, there are microbes that are important to sulfur cycling. Typically in anaerobic subsurface sediments, sulfate reducing bacteria (SRB) use sulfate as a terminal electron acceptor instead of oxygen, resulting in sulfide compounds (Hansen, 1994). We found multiple SRB taxa within the family Thermodesulfovibrionaceae and the genus *Desulfococcus* that distinguish sediment from other microhabitats (Table 2; Galushko and Kuever, 2019; Umezawa et al., 2021). Then, much of the sulfide formed by SRB in sediments is oxidized back to sulfate by sulfur-oxidizing bacteria (SOB; Jørgensen and Nelson, 2004). Among the bacteria that distinguish shell bacterial communities from other microhabitats, we find SOB in the genera *Novosphingobium* and *Rhodobacter* (Table 2; Imhoff et al., 1984; Imhoff, 2015; Haosagul et al., 2020). We also see the family Comamonadaceae associated more strongly with mussel biodeposits than other microhabitats and this family has also been shown to oxidize sulfur compounds (Zhang et al., 2017). While we detect evidence of both SRB and SOB within the sediment, these SOB distinguish mussel communities from those of the sediment and their differential abundances may be facilitated by mussel activity. It is not unusual in marine sediments for sulfur bacteria to form symbiotic relationships with bivalves that depend on their primary production (Vaughn and Hoellein, 2018). If mussel activity expands the oxic-anoxic interface and delivers SOB to anoxic regions to respire sulfide produced by SRB, this may be an additional and critical mechanism by which mussels influence nutrient dynamics and primary production in freshwater environments. The presence of both SOB and SRB in these environments may prove especially interesting considering the interactions between sulfide produced by SRB and nitrogen removal in streams.

One of the primary mechanisms by which excess nitrogen (N) is removed as N_2_ gas from aquatic ecosystems rather than assimilated into microbial and algal biomass, is through dissimilatory N respiration by microbes in the sediment. However, under laboratory conditions, sulfide has been demonstrated to inhibit enzyme pathways required for both nitrification (Joye and Hollibaugh, 1995) and denitrification (Brunet and Garcia-Gil, 1996; Burgin and Hamilton, 2007). Yet, mussels have been shown to increase the potential for nitrogen removal in sediments proven to have relatively high amounts of sulfur deposition (Newton et al., 2013; Nickerson et al., 2019). When adjusting the ratio of carbon and nitrogen in a bioreactor, dos Santos et al. (2021) found that microbial communities with significantly higher N removal demonstrated increased abundances of six families of bacteria: Saprospiraceae, Chitinophagaceae, Xanthomonadaceae, Comamonadaceae, Bacillaceae, and Planctomycetaceae. We found five of these six families associated with different microhabitats across mussel beds (Tables 2–6) indicating that it may not simply be mussel driven changes in sediment communities that alter nutrient cycling in these systems, but rather interactions between mussel-associated and environmental microbes.

Nitrification and sulfide oxidation occur in oxic sediments while denitrification and sulfate reduction occur in anoxic environments. Mussels often traverse the boundary between oxic and anoxic layers of the sediment and so it is not surprising that we find microbes that encompass these diverse modes of respiration associated with them in the sediment. However, these modes of respiration are complementary, and we find key players in these cycles across both mussels and the sediment. These patterns suggest that the role of burrowing bivalves in facilitating interactions between these microbes may be underestimated. Further, delivery of both S contaminants and nitrates to anaerobic sediments can enhance both sulfide oxidation and denitrification (Cardoso et al., 1996) and sulfur cycling by microbes has been shown to account for a large portion of nitrate removal in streams, lakes, and wetlands (Burgin and Hamilton, 2008). Mussels may provide a niche for microbes with a wide array of respiratory functions that in summation serve to remove contaminants of ecological concern (Turner and Rabalais, 2003; Burgin and Hamilton, 2007).

## Conclusion

In our exploratory study of the microbiomes of mussels (shell and biodeposits) and the environment they inhabitat (sediment) we found that mussel microbiomes were less diverse than those of the sediment, mussel abundance was a significant predictor of sediment microbial community composition, and local habitat influenced microbial assemblage composition more than site spatial location along the river. Our findings indicate that rather than a continuous shift in beta diversity along the river, microbial communities further away from each other are more similar than communities next to each other. In our system, a regional pool of bacterial taxa may thus be filtered by site-specific environmental conditions along the river continuum. Further, the presence of macro-organisms may be an additional mechanism that shapes the microbiome of benthic communities. Although synergistic communities of microbes are likely to persist in the sediment, interactions may be limited. Animal-facilitated interactions between freshwater microhabitats have implications for the removal of environmentally impactful metabolites such as nitrates and sulfides. We suggest more thorough testing of the impact of mussels and other burrowing organisms on microbial community diversity and function. Mussel aggregations may provide a niche for microbial communities that undoubtedly play a role in the complex cycling of multiple nutrients critical to primary production. Alterations to nitrogen and sulfide removal become especially important when considering anthropogenic inputs into freshwater systems.

## Data Availability Statement

Data are available through Open Science Framework: Vaughn et al. (2021).

## Author Contributions

EH, TP, and CV designed the comparative field study. EH performed analyses and wrote the initial manuscript with subsequent input from all authors.

## Conflict of Interest

The authors declare that the research was conducted in the absence of any commercial or financial relationships that could be construed as a potential conflict of interest.

## Publisher’s Note

All claims expressed in this article are solely those of the authors and do not necessarily represent those of their affiliated organizations, or those of the publisher, the editors and the reviewers. Any product that may be evaluated in this article, or claim that may be made by its manufacturer, is not guaranteed or endorsed by the publisher.
